# Validating Intravascular Imaging with Serial Optical Coherence Tomography and Confocal Fluorescence Microscopy

**DOI:** 10.3390/ijms17122110

**Published:** 2016-12-15

**Authors:** Pier-Luc Tardif, Marie-Jeanne Bertrand, Maxime Abran, Alexandre Castonguay, Joël Lefebvre, Barbara E. Stähli, Nolwenn Merlet, Teodora Mihalache-Avram, Pascale Geoffroy, Mélanie Mecteau, David Busseuil, Feng Ni, Abedelnasser Abulrob, Éric Rhéaume, Philippe L’Allier, Jean-Claude Tardif, Frédéric Lesage

**Affiliations:** 1 Département de Génie Électrique et Institut de Génie Biomédical, École Polytechnique de Montréal, Montreal, QC H3T 1J4, Canada; maxime.abran@gmail.com (M.A.); alexandre.castonguay87@gmail.com (A.C.); joel.lefebvre@gmail.com (J.L.); 2 Montreal Heart Institute, Montreal, QC H1T 1C8, Canada; mariejeanne.bertrand@gmail.com (M.-J.B.); barbarastaehli@hotmail.com (B.E.S.); nolwenn.merlet@gmail.com (N.M.); Teodora.Mihalache-Avram@icm-mhi.org (T.M.-A.); Pascale.Geoffroy@icm-mhi.org (P.G.); melanie.mecteau@icm-mhi.org (M.M.); david.busseuil@icm-biobanque.org (D.B.); Eric.Rheaume@icm-mhi.org (É.R.); philippe.l_lallier@icloud.com (P.L.); jean-claude.tardif@icm-mhi.org (J.-C.T.); 3 Département de Médecine, Université de Montréal, Montreal, QC H3C 3J7, Canada; 4 National Research Council Canada (NRCC), Montreal, QC H3A 1A3, Canada; Feng.Ni@cnrc-nrc.gc.ca (F.N.); abedelnasser.abulrob@nrc-cnrc.gc.ca (A.A.)

**Keywords:** intravascular ultrasound (IVUS), near-infrared fluorescence (NIRF), atherosclerosis, ex vivo three-dimensional (3D) histology, optical coherence tomography (OCT), confocal fluorescence microscopy

## Abstract

Atherosclerotic cardiovascular diseases are characterized by the formation of a plaque in the arterial wall. Intravascular ultrasound (IVUS) provides high-resolution images allowing delineation of atherosclerotic plaques. When combined with near infrared fluorescence (NIRF), the plaque can also be studied at a molecular level with a large variety of biomarkers. In this work, we present a system enabling automated volumetric histology imaging of excised aortas that can spatially correlate results with combined IVUS/NIRF imaging of lipid-rich atheroma in cholesterol-fed rabbits. Pullbacks in the rabbit aortas were performed with a dual modality IVUS/NIRF catheter developed by our group. Ex vivo three-dimensional (3D) histology was performed combining optical coherence tomography (OCT) and confocal fluorescence microscopy, providing high-resolution anatomical and molecular information, respectively, to validate in vivo findings. The microscope was combined with a serial slicer allowing for the imaging of the whole vessel automatically. Colocalization of in vivo and ex vivo results is demonstrated. Slices can then be recovered to be tested in conventional histology.

## 1. Introduction

Atherosclerosis is a chronic immune-mediated inflammatory disease that arises from a series of complex events triggered by endothelial dysfunction, lipid accumulation in the arterial wall, and infiltration of monocyte-derived macrophages [1,2]. Acute coronary syndromes (ACS) occur mostly from the rupture of modestly stenotic lipid-rich “vulnerable” plaques, which leads to endoluminal thrombus formation, myocardial ischemia, and sudden cardiac death [3,4]. Although coronary angiography remains the gold standard for epicardial coronary stenoses assessment and treatment, it frequently underestimates true plaque burden and provides no information regarding plaque composition [5]. Intravascular ultrasound (IVUS) imaging has been established as an adjunct imaging technology to coronary angiography, widely used in both clinical and research applications [6]. By generating in vivo cross-sectional images of the vessel wall and lumen, IVUS enables the characterization of atherosclerotic vessel segments by providing accurate lumen and vessel dimensions, as well as non-protruding plaques, positive vascular remodeling, and plaque burden assessment [7,8]. Conventional grayscale IVUS is, however, limited with regards to the analysis of plaque composition [9], whereas emerging molecular imaging technologies, such as fluorescence imaging, have been developed to overcome these limitations. Multimodality imaging systems, such as the dual-modality IVUS/near-infrared fluorescence (NIRF) imaging catheter previously engineered by our group and others [10,11,12,13], were designed for integrated microstructural and molecular plaque imaging, thus enabling a more detailed plaque characterization. The use of molecular probes in conjunction with fluorescence imaging has been shown to provide complementary information with regards to plaque activity and inflammation [14,15,16,17,18,19]. Translation of molecular imaging results to clinical applications, however, requires validation; and despite impressive advances in intravascular imaging over the past decade, histology remains the gold standard for determining plaque composition and geometry. Although providing high-resolution cross-sectional images of the arterial wall, histology remains limited to the number of tissue sections analyzed and by the lack of anatomical context; thus resulting in missed valuable data. When comparing in vivo intravascular imaging applications with histology, colocalization is often challenged by geometric distortions and tissue shrinkage, as well as the lack of anatomical landmarks and the limited resolution of IVUS imaging.

OCT-based block-face three-dimensional (3D) histology combined with serial cutting of tissues has been proven in the past to be an efficient technique to reconstruct and visualize whole intact organs or tissues [20,21]. Previous work has demonstrated the use of serial OCT imaging primarily for brain imaging. However, this method has so far never been used for cardiovascular imaging. From the spatial resolution of optical coherence tomography (OCT), largely superior to IVUS [6], and the capacity of confocal fluorescence microscopy to efficiently identify the same molecular biomarkers as NIRF imaging [22], we developed a novel ex vivo automated 3D histology platform comprising a dual-modality imaging system based on OCT-coupled fluorescence sensitive confocal microscopy [21]. In this work we detail the process of image reconstruction using this system and, for the first time, describe its use for the purpose of atherosclerosis detection and localization in iliac arteries and aortas of an atherosclerotic rabbit model. Fluorescent signal colocalization obtained from in vivo and ex vivo imaging was performed to validate the potential of these methods to be co-registered and for OCT-combined fluorescence sensitive confocal microscopy to serve as a future histology add-on validation tool in the development of novel molecular probes.

## 2. Results

### 2.1. In Vitro Affinity of Anti-ICAM-1 Antibody

**Figure 1:**
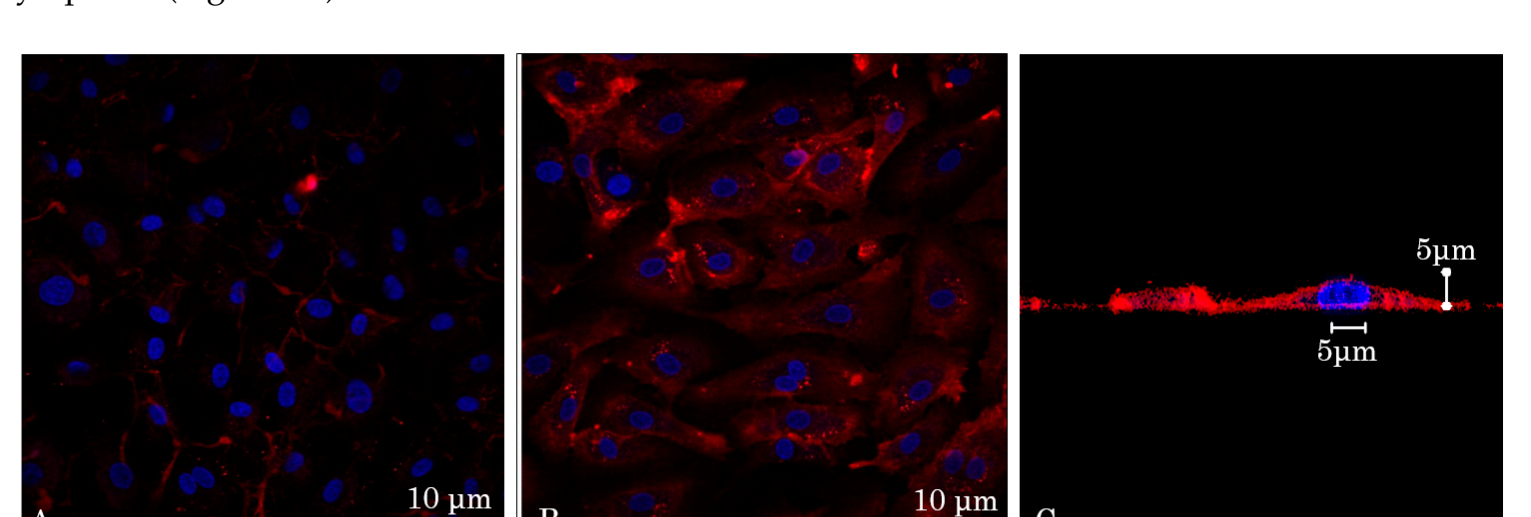
Florescent confocal images of in vitro affinity of the intercellular adhesion molecule-1 (ICAM-1) antibody: (**A**) Inactivated and (**B**) Tumor Necrosis Factor Alpha (TNF-α) activated Human Umbilical Vein Cells (HUVEC). The nuclei (blue) were stained with DAPI (4′,6-Diamidino-2-Phenylindole, Dilactate) (**C**) deconvolved image of a cell. The fluorescence signal (red) is not present in the cytoplasm or the nucleus, but is, rather, located on the cell membrane.

A fluorescently labelled anti-intercellular adhesion molecule-1 (ICAM-1) antibody was used as a marker of inflammation below. Four ICAM-1 probes were initially tested in vitro, but only one showed positive affinity with inflammation (Figure 1). Fluorescence confocal microscopy images were taken to evaluate the affinity of the anti-ICAM-1 antibody with mammalian cells. Human Umbilical Vein Cells (HUVEC) were imaged before and after being activated by Tumor Necrosis Factor Alpha (TNF-alpha), which induces inflammation. Placing the fluorophore bound to the ICAM-1 antibody in the cell growth medium followed by flushing, it was observed that the signal was far more present for the TNF-alpha activated cells, suggesting that the ICAM-1 antibody does in fact have affinity with inflammation (Figure 1). Deconvolution was also performed to form a transverse image of the cell to show that the signal was localized on the cellular membrane and not in the growth medium or the cytoplasm (Figure 1C).

### 2.2. In Vivo Catheter Imaging

**Figure 2:**
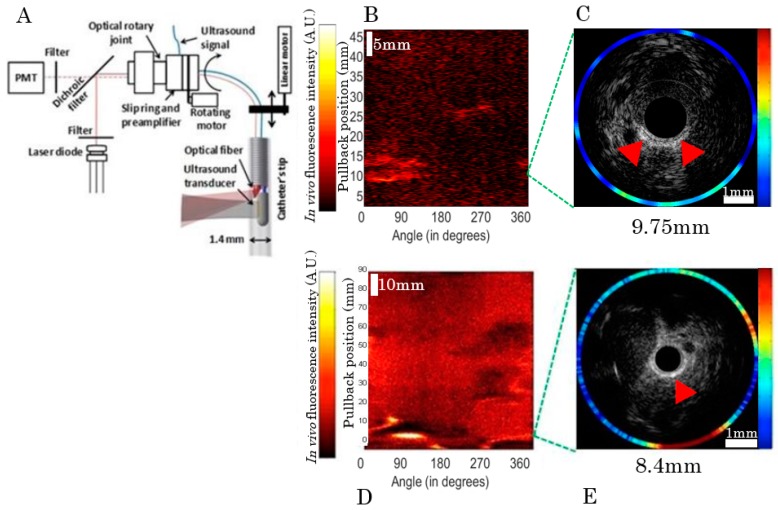
In vivo imaging system and typical images. (**A**) Overview of the bimodal catheter system with a detailed view of the catheter’s tip. PMT stands for photomultiplier tube; (**B**) Paired in vivo near-infrared fluorescence signal detected over 360 degrees with 50 mm pullback length; and (**C**) integrated NIRF-IVUS cross-sectional imaging with partial fluorescence signal and echolucent plaque colocalization (shown by red arrows) in model 1. Atherosclerotic plaque, shown by echolucent signal on IVUS (**D**,**E**), was partly correlated with indocyanine green (ICG)-fluorescence signal at 8.4 mm of pullback (red arrow) in model 2.

Five atherosclerotic rabbits were imaged following either an in vivo targeted molecular probes injection (model 1) or an intravenous indocyanine green (ICG) (model 2) injection with a dual IVUS/NIRF imaging catheter designed by our group (Figure 2). The IVUS had a frequency of 45 MHz and the excitation wavelength of the NIRF was 780 nm. As shown in Figure 2B,C, a strong in vivo signal was obtained following injection of an ICAM-1 nanobody probe at 30.05 mm of pullback in model 1, with partial correlation with the echolucent region on IVUS imaging. Other ICAM-1 nanobodies that did not show affinity in vitro were also injected with the purpose of evaluating their targeting ability. For these, weak signals were detected by in vivo NIRF/IVUS imaging, despite the presence of plaque on IVUS. In model 2, a strong but localized signal was seen at 8.4 mm of catheter pullback correlated with IVUS imaging, with the plaque pointed by the red arrow (Figure 2E). The pulse generated by the ultrasound (US), created an artifact and a mask was applied to hide the catheter on the images up to the artifact radial position, which extended 50 µm past the wall of the catheter. Thus, part of the mask intersected the vessel wall (Figure 2E) in smaller vessels, but not in bigger ones (Figure 2C). A ring artifact that can be seen of Figure 2C was caused by a reflection on the sheath surrounding the catheter.

### 2.3. Serial Imaging System, Ex Vivo OCT Reconstructions and Fluorescence Alignment

**Figure 3:**
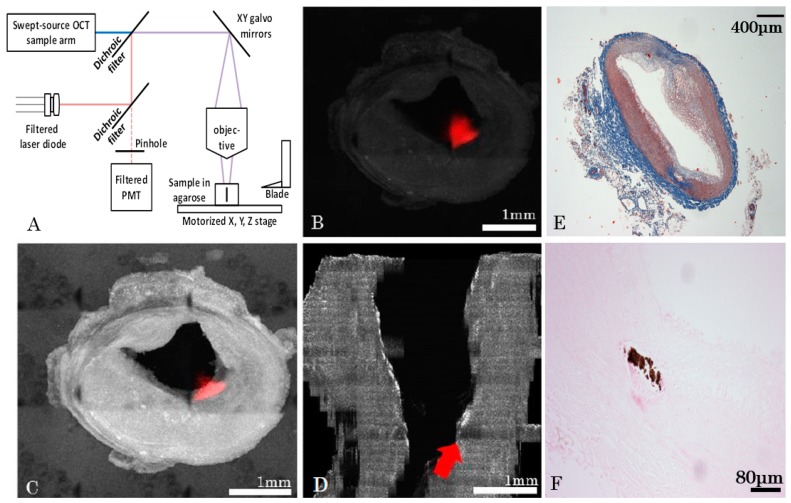
Ex vivo optical coherence tomography (OCT) and confocal reconstruction for a model 1-type rabbit. (**A**) Overview of the serial histology imaging system; (**B**) Example of an averaged slice (raw data) with the fluorescence image superimposed on it (i.e., red signal); (**C**) Example of the same slice after Beer-Lambert corrections, contrast adjustment, and deconvolution (Log-scale), with superimposed fluorescence image (red); (**D**) Localization of the tissue slice (c) on a 3D reconstruction (shown by red arrow); (**E**) Histology slice colored with Masson Trichrome and a 4× objective (**F**) Same slice imaged with VonKossa and a 20× objective.

A serial microscopy imaging system was designed combining OCT and confocal fluorescence imaging (Figure 3A). Micrometer-precise motors and a razor blade attached to a custom vibratome allowed automatic serial mosaic imaging of agarose-embedded artery sections to be performed. OCT and fluorescence data were measured simultaneously which enabled co-registration of OCT imaging with fluorescence. OCT provided 3D volumes for each slice (200 µm thickness) while a single confocal image was taken (2D) with focus centered in the slice. For confocal fluorescence, a trade-off was required in the system to enable the combination of OCT (which requires a long focal depth for accurate 3D images) and confocal microscopy requiring filling the objective for optimal resolution. In this design, the confocal beam under-filled the objective, leading to a fluorescent point-spread-function that extended in depth.

The model 1 rabbits were imaged with a 3× objective whereas the model 2 rabbit was imaged with a 10× water immersion objective. The former being designed to work in air, a chamber was built between the end of the objective and water by gluing a glass window to the aluminum lens tube. Typical results are presented in Figure 3B–D for raw, processed, and 3D reconstruction data, respectively. Fluorescence signal was higher at the surface or near the arterial wall, which suggests that the ICAM-1 nanobodies bind to lipid plaques and that these molecular probes are sources of specific signals. The processed slices can then be recovered after slicing and imaged in standard histology (Figure 3E,F), here, with Masson Trichrome (Figure 2E) and Von Kossa (Figure 3F) to detect calcification.

#### Data Reconstruction

Due to tissue attenuation of the OCT signal and serial mosaic acquisitions, specific algorithms were developed to reconstruct full 3D volumes. A custom Python stitching and signal correction algorithm was developed, allowing complete artery reconstructions and atherosclerosis localization based on both modalities. The following steps were implemented:

- For each tissue slice, the volume position in the mosaic reference frame was estimated using microscope acquisition data;
- Volumes were then stitched for each tissue slice;
- Post processing was performed that included cropping of the field of view, identification of lumen mask and Beer-Lambert intensity correction;
- After post processing steps, slices were assembled together in order to form a longitudinal 3D volume.

**Figure 4:**
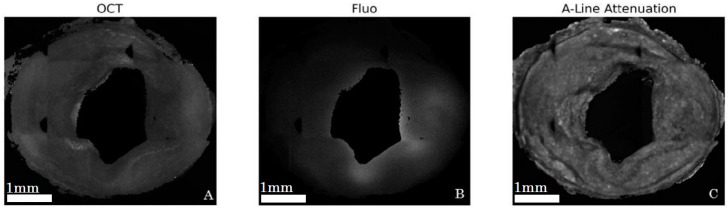
Corrected images for model 1 rabbit for OCT (**A**); and fluorescence (**B**); A-line attenuation map used for OCT intensity correction outlines detailed tissue structures (**C**).

Figure 4A,B show typical images after the post processing steps (masks, Beer-Lambert correction, intensity artifacts correction, and cropping) applied to an averaged OCT slice and fluorescence image, respectively. Figure 4C shows the attenuation map used for Beer-Lambert correction for a particular slice.

As previously mentioned, with an under-filled objective used in fluorescence imaging, deeper tissue slices could contaminate the signal of the imaged slice in fluorescence due to the extended point-spread function (PSF), thus, requiring a deconvolution algorithm to generate precise fluorescence images. A synthetic PSF was generated using the PSF generator plugin in the ImageJ software (National Institutes of Health, New York, NY, USA) using the Born and Wolf 3D optical model. It had a FWHM of 4.4 µm in the *x*-*y* direction and of 146 µm in the *z* direction and was used to correct fluorescence images.

**Figure 5:**
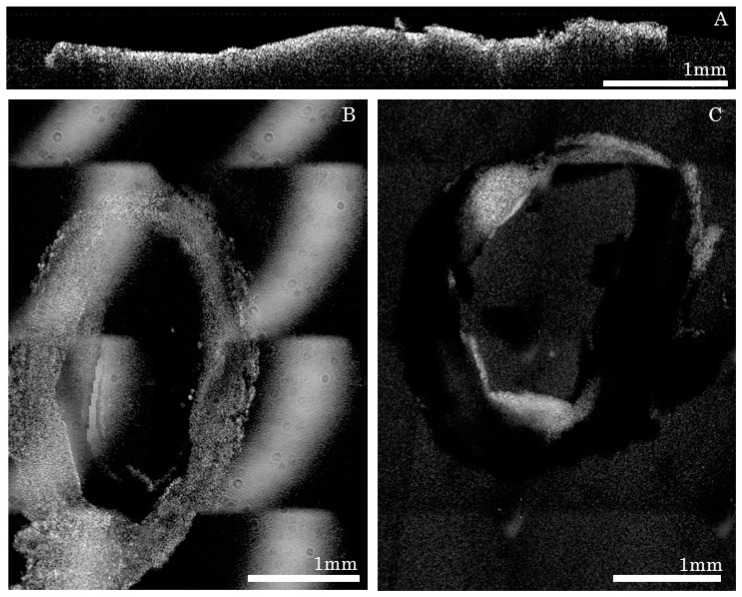
Sources of imaging artifacts and their effects during acquisitions: (**A**) Unevenly cut slice; (**B**) Artifact caused by the glass when the reference arm was not properly placed; (**C**) Slice that was imaged while not placed at the focal point of the lens.

When comparing to brain imaging obtained from a similar technology, the automatic ex vivo imaging technique required careful preparation of arterial tissues, as conjunctive tissues could cause cutting artifacts (Figure 5A), thus making it challenging to obtain uniform cuts. An algorithm was applied during image acquisitions to ensure fine control of the focal depth and to avoid placing tissues in areas where OCT had instrumental artifacts (spurious reflections) or outside the focal zone of the objective (Figure 5B,C).

### 2.4. Alignment and Tissue Deformation

**Figure 6:**
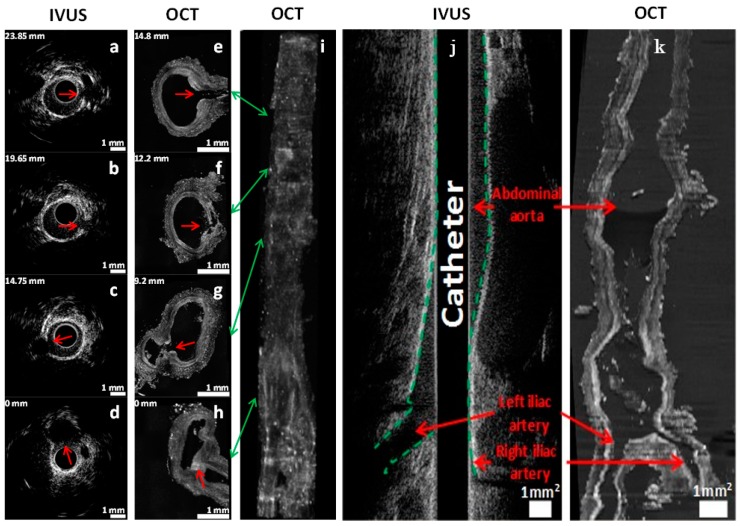
Intravascular ultrasound (IVUS) and OCT colocalization of anatomical landmarks in model 2. (**a**–**d**) In vivo IVUS cross-sectional images; (**e**–**h**) Ex vivo OCT cross-sectional images; (**i**) 3D reconstruction in OCT using a maximum intensity projection algorithm. Indicated numbers in mm (upper left of each image) represent the distances between the cross-section and the iliac bifurcation. The catheter was introduced in the right iliac artery, located at the bottom-right in the OCT image in (**h**,**i**). Green arrows indicate the location of the cross-section slices on 3D reconstruction. Red arrows denote side branches (anatomical landmarks) used for colocalization. Longitudinal view of the abdominal aorta and iliac arteries in IVUS and OCT imaging in model 2; (**j**) In vivo IVUS image of a 50 mm artery segment (green dashed lines delineates the arterial wall); (**k**) Ex vivo OCT image of the same segment, which shrunk to a length of 30 mm after ex vivo tissue fixation. Scale bars represent a region of 1 mm by 1 mm.

Due to ex vivo tissue fixation and a lack of intra-arterial pressure, which led to tissue dehydration and shrinkage, the comparison of in vivo and corresponding ex vivo vessel segments was challenging. Despite the average tissue shrinkage ratio of 61% that was calculated in our experiments, imaging colocalization was possible using landmarks. Longitudinal views of both IVUS and OCT anatomical imaging of an arterial segment are presented in Figure 6. The abdominal aorta and iliac arteries were visualized with both modalities, which served as reference points for colocalization. While longitudinal co-registration was possible, precise pixel-wise deformation models could not be applied since the arterial wall was highly distorted in ex vivo OCT images given the lack of blood flow in fixed tissues. Nevertheless, longitudinal segments could be identified accurately, which enabled comparisons of pullback in vivo results to ex vivo data.

### 2.5. Validation of Intravascular Molecular Imaging

**Figure 7:**
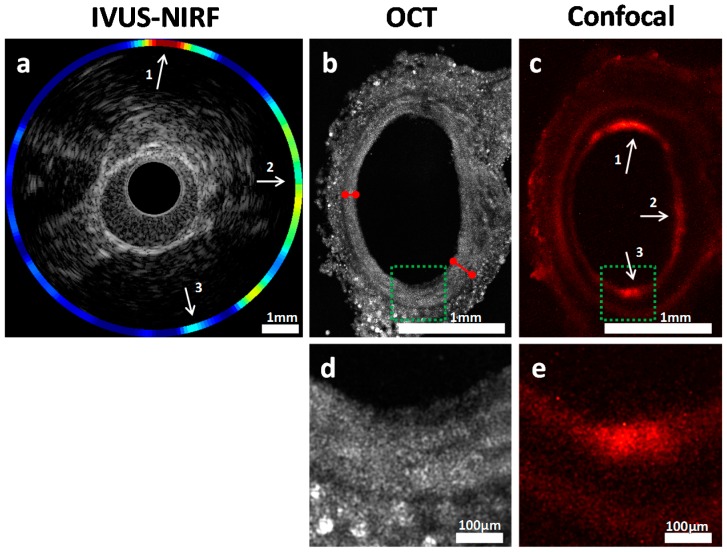
Cross-sectional view of the abdominal aorta in model 2. (**a**) IVUS-NIRF imaging in vivo; (**b**) OCT imaging ex vivo; (**c**) Confocal fluorescence microscopy imaging ex vivo; (**d**,**e**) Enlarged sections of the green region of (**b**,**c**). Red arrows identify the intimal thickness at two locations in the OCT image. White arrows indicate colocalization between in vivo and ex vivo fluorescence. Arrow 3 shows a weaker colocalization due to the limitation of our blood attenuation compensation algorithm [19], further supporting the need for co-registered ex vivo validation.

Using the methodological steps outlined above, in vivo ICG accumulation identified with NIRF imaging was confirmed using high-resolution fluorescence confocal imaging, as shown in Figure 7. Intimal thickening was also observed on ex vivo OCT, an indication of the presence of plaque. Figure 7b shows that the intimal thickness varied from 100 µm to 200 µm (red arrows), a difference not perceptible in IVUS, which has a resolution of about 100 µm.

## 3. Discussion

Combining IVUS and fluorescence imaging (NIRF) within a single catheter may yield a reliable method that could be used to detect and locate atherosclerotic plaques in the arterial wall. This imaging method overcomes the shortcomings of coronary angiography, whereas fluorescence provides information regarding plaque composition and biology. Translation of such imaging technology to clinical applications requires robust preclinical validation, and co-registration of in vivo data to ex vivo assessments is essential for better plaque assessment. In the present work, we demonstrated that multimodal custom serial imaging of tissue sections can be used to corroborate in vivo findings using OCT and confocal imaging to provide high spatial resolution. Our system is also compatible with conventional histology, as the slices from the vibratome can be recovered in the correct order using a small container placed in front of the agarose block and stained using standard procedures.

The main shortcoming of conventional histology is that although it offers information on molecular tissue composition, it does not provide accurate anatomical localization of said tissues. There is, at the moment, no existing method that can reliably corroborate the in vivo plaque localization and composition measurements done with an intravascular catheter during pullbacks. The method described here can, however, combine the molecular composition determination aspect of conventional histology, while the 3D reconstructions allow colocalization to be performed. Furthermore, in conventional histology, images are formed after the tissue is sliced, which leads to additional deformations and difficulties in reconstructing the 3D view. With the block-face OCT technique, images are taken before slicing, facilitating 3D assembly. Our method, while not a substitute for standard histology, is projected to be a complementary method that can help to bridge in vivo and histological data. The ability to co-localize tissues between the in vivo and ex vivo tissues offers a novel approach that could be used to validate future intravascular molecular imaging studies. Having a reliable way to confirm the data gathered in in vivo scans will smooth the transition between the fundamental research and clinical domains, leading to more effective, invasive imagery techniques and eventually better treatments for patients.

### 3.1. Slicing Optimization

One of the most challenging aspect of the ex vivo imaging process was to obtain uniform flat slices with the vibratome, as the connective tissue surrounding the artery rendered slicing more difficult. Residual connective tissue remaining above the sample after slicing appeared to block the light and/or cause inaccurate surface detection, thus, leading to some slices being imaged while being out of focus. Furthermore, slice stitching in the *z*-direction was very sensitive to the flatness of the slices. Keeping the slicer blade completely flat and straight at all times was difficult and resulted in different slice thicknesses. Crooked or uneven slices were problematic during reconstructions, since stitching was based on surface detection. To overcome these difficulties, a fixed overlap between slices was imposed. However, this could create gaps, which sometimes led to the presence of dark bands between slices in the longitudinal reconstruction of the aorta. It should be noted that those overlaps may also induce a small bias when approximating tissue shrinkage as the z-stitching was highly dependent on these. Finally, evaluating the shrinking factor was also made more difficult by the fact that our method relied on finding landmarks, which were sometimes sparse. Optimizing tissue embedding and a careful removal of connective tissue is, thus, key to gathering quality data.

### 3.2. Optical Optimization

In the case of the 3× objective, the glass used to protect it from water where the sample was imaged was a source of artifacts, since a reflection and its harmonics could be seen with the OCT scanner. While adjusting the reference arm’s length could minimize the effect of the glass by imaging in the opportune zones, it was never possible to fully remove its effects since the automatic imaging process, which takes a few days to perform, always yielded a few images that were not situated in the ideal zone. Since the glass has very high reflectivity, the artifact often saturated the detector and information was lost when it could be observed within the imaged tissue. It was, thus, very important for the experimenter to place the glass at a position that did not create an artifact near the focal point of the objective.

### 3.3. Big-Data Processing

The size of the datasets acquired was a factor that rendered data analysis quite cumbersome. A raw dataset of 500 GB for a single artery section required significant computing power to process and disk space for storage. Even lowering the resolution by going from a 10× to a 3× objective, the image sizes only decreased by half. Improvement in processing in terms of efficiency and storage will be required to enable large-scale studies.

## 4. Materials and Methods

### 4.1. Animal Model for Atherosclerosis

Six adult, male, New Zealand White rabbits (3–4 kg, 3 months old; Charles River Laboratories, Saint-Constant, QC, Canada) were fed a 0.5% cholesterol diet (Harlan Techlab Diets, Madison, WI, USA) to induce atherosclerosis. Two rabbit models were used: model 1 (*n* = 5), balloon injury performed at week 0, followed by 14-weeks cholesterol-enriched diet; model 2 (*n* = 1), 14-weeks of high-cholesterol diet. One rabbit did not complete the study due to neurologic complications after completion of balloon denudation. The experimental protocols were approved by the animal ethics committee of the Montreal Heart Institute Research Center (Projet ID code: 2015-1827, 2015-32-02, accepted on 24 March 2015) according to the guidelines of the Canadian Council on Animal Care.

### 4.2. Balloon Dilatation Procedures

Under general anesthesia (ketamine (35 mg/kg) and buprenorphine (10 mg/kg) administered intramuscularly, inhaled isoflurane (3% *v*/*v*, Baxter, Deerfield, IL, USA) and supplemental oxygen), balloon injury of the abdominal arterial wall was performed using a 5 French (F) radial introducer catheter (Cordis Corporation, Fremont, CA, USA) introduced through the right carotid artery. Under fluoroscopic guidance (Siemens, Berlin, Germany), a 4.0 mm PTCA balloon catheter (Boston Scientific, Marlborough, MA, USA) was advanced at the iliac bifurcation over a 0.014 inch guidewire (Abbott Vascular, Santa Clara, CA, USA), inflated with 8 atm and retracted three times in the distal 40 mm of the abdominal aorta. The right carotid artery was ligated at the end of the procedure.

### 4.3. Bimodal Near-Infrared Fluorescence (NIRF)/Intravascular Ultrasound (IVUS) Imaging Catheter System

The in vivo imaging system (Figure 3A) used in this study, designed as an ultrasound-optical imaging catheter linked to an optical assembly and custom-made electronics, was previously described in [10]. The catheter combines an optical fiber for fluorescence imaging and an ultrasound transducer for acoustic imaging. An electronic circuit synchronizes the acquisition with two motors driving the rotating/translating catheter assembly, and raw data is transferred directly to a laptop via a universal serial bus (USB) connection at rates of up to 250 Mbps. A custom Matlab (The MathWorks, Inc., Natick, MA, USA) user interface filters the signals and reconstructs and displays the images in real time during acquisition. Fluorescence excitation was performed using a 780 nm laser diode and emission was detected by a photomultiplier tube (Hamamatsu Photonics, Hamamatsu City, Japan), combined with a bandpass optical filter (832 ± 19 nm). A compensation algorithm was used to adjust the fluorescence signal amplitude for blood attenuation [10].

### 4.4. In Vivo NIRF Imaging Procedure

In vivo NIRF imaging was performed under anesthesia, as previously described, using a 5 F introducer (Cordis Corporation, Fremont, CA, USA) placed in the left carotid artery (model 1; *n* = 4). Four novel tentative imaging probes (nanobodies) targeting ICAM-1 receptors (National Research Council Canada, Ottawa, CA, USA), labeled with infrared dye 800CW, were injected under fluoroscopic guidance (Siemens, Germany) in the denuded segment of the abdominal aorta of model 1, followed by intravascular IVUS/NIRF imaging. Intravenous indocyanine green (ICG, 10 mg/kg) was injected in model 2 (*n* = 1) and intravascular imaging was performing through a right carotid artery access 40 min after dye injection. Automated imaging pullbacks were performed in the abdominal aorta of both animal models and in the right iliac artery of model 2 at a pullback speed of 0.5 mm/s and a frame rate of 10 images/s over a total length of 50 and 100 mm, respectively. After in vivo imaging procedures, the animals were sacrificed by exsanguination under anesthesia and then underwent abdominal aorta and iliac arteries resection. The samples were fixed in 4% formaldehyde and kept at 4 °C.

### 4.5. Ex Vivo Imaging System and Methods

Prior to ex vivo imaging, the distal 40 mm of the abdominal aorta and iliac arteries were mounted in a 4% cylindrical agarose block with 0.5% ethylenediaminetetraacetic acid (EDTA). Ex vivo imaging was performed using a custom made automated serialized dual-modality setup for OCT and confocal fluorescence microscopy, incorporating a swept-source laser with a central wavelength of 1310 nm and a tuning bandwidth of 100 nm for OCT. The confocal laser passed through a filter cube before being reflected at a 90° angle by a long pass dichroic mirror at 875 nm which allowed for it to be combined with the OCT laser beam (Figure 3), allowing simultaneous acquisition of OCT and confocal data. To overcome limited light penetration in the tissue and scattering from microscopic imaging, a vibratome allowed sequential sectioning of the tissue block face in order to reveal new tissue regions to image. Following a previously described design, a dual flexure part isolated vibration of the blade from a direct current (DC) motor on a one-axis yielding precise cutting of the tissue. X and Y stages allowed moving the sample relative to the objective and imaging every sub-region (1 by 1 mm). Tissue samples embedded in agarose blocks were placed underneath a 10× water immersion objective for model 2. After an entire section was imaged, a 200-µm slice of tissue was removed and the process was repeated automatically. For each slice, the Z position of the sample was adjusted in order to have the focus approximately 50 µm under the tissue surface, thus avoiding imaging of deformed tissue due to its slicing. The images from both in vivo and ex vivo systems were analyzed using Matlab. Anatomical landmarks allowed for a precise sub-millimeter colocalization of in vivo and ex vivo images, along with the calculation of the shrinkage ratio of the excised tissue. To obtain three-dimensional (3D) reconstructions, imaging data was downsampled to a voxel size of 4 µm by 4 µm by 200 µm, converted in DICOM and loaded with Osirix (Pixmeo, Geneva, Switzerland). A maximum intensity projection algorithm was applied to generate a three-dimensional view of the vessels. The ex vivo imaging system generated cross-sectional OCT image slices with a pixel size of 2 µm by 2 µm and a depth of 6.5 µm. The resulting dataset had a size of over 500 GB. In fluorescence confocal microscopy, one cross-sectional image was obtained for each 200-µm depth. Colocalization between the IVUS and OCT images was performed. Figure 6 compares the OCT reconstruction with the IVUS scan for rabbit 1. Colocalization between images relied on finding biological landmarks, primarily bifurcations. Pullbacks began at the bifurcation between the left and right iliac artery and could be seen on both types of scans, providing a good starting point.

For model 1, aortas were imaged using the aforementioned OCT/confocal microscope, but with a 3× air objective and a 5 µm × 5 µm pixel size. Axial resolution was unchanged. A glass was added between the objective and the water to create an air chamber. The field of view (FOV) in the lateral directions was set to 2.5 mm × 2.5 mm. The ICAM-1 fluorophore was sensitive to a 776 nm wavelength. The 3× objective did not allow for a high enough signal-to-noise ratio (SNR) with a small pinhole (100 µm), and an iris was used instead. This, however, came at the cost of lateral resolution (4.4 µm). The vibratome blade was inclined at an approximately 20-degree angle with the plane of the agarose gel. Connective tissue was removed before embedding the aorta in agarose with a razor blade. Three-dimensional reconstructions of the aortas were done using custom Python algorithms developed for this purpose. Since an iris was used instead of a pinhole, deconvolution was performed on the fluorescence images in order to locate more accurately the sources of signal.

For OCT images, the first reconstruction step was to find the volumes position within the mosaic reference frame. A displacement model of the sample-motorized stage was used to estimate each XY tile position within the mosaic. The model parameters were estimated from the data by computing the phase-correlation based pairwise registration [23] of all neighboring tiles within the mosaic and by inverting the model, solving for each parameter. The next step was to stitch together the volumes for each slice. Adjacent volumes were blended together by finding the medial axis of their 2D overlap region and by applying a small Gaussian feathering to ensure a smooth transition between tiles. Each tissue slice was stitched separately.

A few post processing steps were then applied on each slice to remove intensity artifacts, to limit the field of view to the tissue, to compute the lumen mask, and, finally, to estimate and compensate the light attenuation with depth in the OCT volumes. This last step was done by fitting a Beer-Lambert law on each A-Line and by estimating the attenuation coefficient from this fit [24]. The Beer-Lambert law was then used again with the average tissue attenuation coefficient to normalize the OCT volume and thus reduce its contrast variation with depth.

The last reconstruction step was to stitch the slices together in the *z* direction to get a complete 3D volume. This was done by computing the shift between adjacent slices using the cross-correlation of their 2D image gradient magnitude. Then, the slices were stitched together by solving the Laplace equation with Dirichlet boundary conditions over their masked overlap region. The tissue mask was used to remove the gaps introduced by the slice cutting artifacts.

Colocalization between the IVUS and OCT images was performed. Colocalization between images relied on finding biological landmarks, primarily vessel bifurcations. Pullbacks began at the bifurcation between the left and right iliac artery and could be seen on both types of scans for model 2, providing a good starting point. The distance between the same landmarks was measured by knowing the pixel size (6.5 µm) for OCT, and by comparing it to the distance found on the IVUS data, which was recorded during acquisition.

## 5. Conclusions

In conclusion, a method for atherosclerotic plaque detection and molecular characterization has been investigated both in vivo and ex vivo in a rabbit model. The in vivo method relied on an intravascular catheter that combined IVUS and fluorescence imaging, while the ex vivo method combined an OCT and a fluorescence confocal microscope with a custom serial slicer and stitching algorithm to reconstruct whole 3D segments of aortas and locate the presence of plaque with great accuracy. Colocalization between the in vivo and ex vivo data was performed by finding landmarks between the IVUS and OCT volumes. This massive histology method is a promising approach to validating future intravascular catheters; it could potentially become a new gold standard to validate intravascular molecular imaging, and it is a great addition to the currently used histology methods.
